# Trends in multiplicity of *Plasmodium falciparum* infections among asymptomatic residents in the middle belt of Ghana

**DOI:** 10.1186/1475-2875-12-22

**Published:** 2013-01-17

**Authors:** Akua Agyeman-Budu, Charles Brown, George Adjei, Mohammed Adams, David Dosoo, Dominic Dery, Michael Wilson, Kwaku P Asante, Brian Greenwood, Seth Owusu-Agyei

**Affiliations:** 1 Kintampo Health Research Centre, Ghana Health Service, Ministry of Health, PO Box 200, Kintampo, Ghana; 2 College of Health Sciences, University of Ghana, Accra, Ghana; 3 Noguchi Memorial Institute for Medical Research, University of Ghana, Accra, Ghana; 4 Faculty of Infectious and Tropical Diseases, London School of Hygiene and Tropical Medicine, London, UK

**Keywords:** Malaria, Multiplicity of infections, Plasmodium falciparum

## Abstract

**Background:**

Malaria is the most important cause of mortality and morbidity in children living in the Kintampo districts in the middle part of Ghana. This study has investigated the multiplicity of infection (MOI) within asymptomatic residents of the Kintampo districts, and the influence of age and seasonality on MOI, by studying the distribution of the polymorphic *Plasmodium falciparum* antigen merozoite surface protein 2 (MSP2).

**Methods:**

DNA was extracted from an asymptomatic cohort of children and adults infected with *P. falciparum* during the period November 2003 to October 2004. Polymerase chain reaction was carried out and multiplicity of infection (MOI) was determined.

**Results:**

Children under 10 years of age had an average MOI of 2.3 while adults 18 years and above had an average MOI of 1.4. Children below five years had high and low average MOIs of 2.8 in the March/April survey and 0.9 in the May/June survey respectively. A similar trend in the monthly distribution of MOI was observed for the entire cohort. IC/3D7 strains outnumbered the FC27 strains throughout the year by a ratio of about 4:1 with the difference between the prevalence of the two strains being least marked in the March/April survey, at the beginning of the rainy season. MOI was not linked to the level of malaria transmission as measured by the entomological inoculation rate.

**Discussion/conclusion:**

The impact of interventions, introduced since this baseline study was carried out on the parasite diversity of asymptomatic residents will be the subject of further investigations.

## Background

Malaria is endemic throughout Ghana and continues to be a major cause of morbidity and mortality. In most parts of Ghana, malaria accounts for over 20% of childhood deaths. According to statistics by the National Malaria Control Programme, about 3.2 million malaria cases are recorded annually resulting in about 38,000 malaria deaths [1].

Epidemiological studies carried out in northern and southern Ghana have documented levels of malaria transmission, bed-net usage, trends in parasite density and severe anaemia, acquisition of parasitaemia in relation to age and sex, and also the prevalence of molecular markers of drug resistance in *Plasmodium falciparum*[2-4]. In the Kintampo districts of Ghana, malaria is a major health problem, which is ranked among the top three causes of mortality and morbidity [1]. Until recently [5], no malaria epidemiological data had been reported from the Kintampo area, which is located in the middle part of Ghana.

The recent reports from Kintampo have shown decreasing parasite densities with increasing age [5,6], amidst entomological inoculation rates (EIR) of 231–269 infective bites per person per year (ib/p/y) [7]. In these reports, EIR, which estimates the level of exposure of an individual to malaria-infected mosquitoes, was used to estimate the malaria transmission rate. In other malaria endemic areas, molecular studies have shown that the number of clones of malaria parasites co-infecting a single host is a useful indicator of transmission intensity, as well as a guide in determining the immune status of the host [8,9]. This view emanates from data, which have shown that an increase in transmission intensity generally correlates with a progressive increase in the average number of malaria parasite clones per host [8,9]. High correlations between parasite density and multiplicity of infection (MOI) have also been reported in infants and young children but not in older individuals [10]. Furthermore, the presence of a high number of clones or MOI is believed to be positively associated with protection against mild episodes of malaria in some circumstances. In holoendemic malarious areas high MOIs have not been related to clinical malaria among older children, [11-13] while in areas of lower transmission, positive relationships between high MOIs and clinical malaria have been observed [14-16].

A study of the multiplicity of malaria infections among an asymptomatic cohort of children and adults resident in the Kintampo districts, based on surveys carried out at two-monthly intervals, over a one-year period, is reported here. This study also examined the distribution of MOI and parasite densities in children less than five years, during different months of the study year as well as the relationships between monthly malaria transmission and merozoite surface protein 2 (MSP2) allelic variants.

## Methods

### Study site

Kintampo North and South districts occupy 18.1% (7,162 km^2^) of the total land area of the Brong-Ahafo Region of Ghana. Located between longitudes 1°25’ and 2°1’’ west of the Greenwich meridian and latitudes 7°43’’ and 8°44’’ north of the equator, the districts’ strategic location makes them the geographical centre of Ghana. The two districts have a resident population of about 140,000 and have a climate, which lies in the transitional zone between the wet semi-equatorial and tropical continental climates. Mean monthly temperatures range between 18°C and 38°C. The area experiences distinct wet and dry seasons, with the wet season beginning in late March and ending in early November each year. Relative humidity in the area is extremely high, varying from between 90% and 95% during the wet season to between 75% and 80% in the dry seasons. Rainfall ranging between 115 cm^3^ and 125 cm^3^ is experienced annually. At the peak of the rainy season, most parts of the districts, especially the northern parts, experience flooding with inundation of vast stretches of land. These conditions provide favourable breeding conditions for mosquito vectors, including the most efficient malaria transmitting vectors, *Anopheles gambiae* and *Anopheles funestus*[7]*.*

### Study design

Results described in this paper were obtained during a large study, which determined the epidemiological patterns and characteristics of *P. falciparum* infections and morbidity from this infection in the Kintampo area as the background for subsequent malaria intervention studies. Details of the main study methodology have been previously reported [6]. The main study started in November 2003 and ended in October 2004. Sampling was carried out among sixteen "index cluster" of contiguous houses, selected on the basis of micro-ecological factors such as water bodies and vegetation types that are known to influence malaria transmission. These sixteen clusters were registered in the database of the Kintampo Health and Demographic Surveillance System, which carries out regular census of all residents and their geo-locations. In-depth details of the main study design have been previously reported [6].

### Ethical consideration

This study was approved by the ethics committee of the Ghana Health Service and the London School of Hygiene & Tropical Medicine.

### Laboratory methods

During the study period, individuals who had provided consent and were asymptomatic for malaria were followed-up on a two-monthly rotational basis; thus, participants were visited six times in the year. Blood samples were collected on each visit. Thick and thin blood smears were obtained and blood blot filtermats (Whatman 3 MM, Whatman, Maidstone, UK) were prepared and labelled for each participant. Thin blood smears (fixed with methanol) and thick blood smears were stained with 10% Giemsa for 10 minutes and examined for *P. falciparum* parasites by oil immersion microscopy. The number of parasites per microlitre was calculated as 40 times the number of parasites counted per 200 leukocytes. At least 100 high power fields were examined before a thick smear was declared negative. Moving averages and proportions were statistically estimated (using StataCorp Stata 9, TX USA) and used to provide descriptions of the parasite density for each participant [5].

In the main study, a minimum number of 140 volunteers were selected in each age group to represent a cluster, to allow an estimated age specific parasite prevalence rates to within 6 -10% of the true values to be calculated for the different age groups [5]. An average of six filter paper samples, determined by microscopy as positive, and representative of the different age categories, was selected from every cluster for each bimonthly survey. Thus, a total of one hundred blood blot filter mats from individuals who were microscopically positive for malaria were selected from the sixteen clusters on each bimonthly survey and a total of 600 samples from the six bimonthly surveys was analysed.

Parasite genomic material was prepared using the chelex method [17]. Genotyping MSP2 (block 3) of *P. falciparum* was performed by amplifying the polymorphic deoxyribonucleic acid (DNA) region of interest using previously described nested polymerase chain reaction (PCR) protocols [18,19]. Primary PCR primers corresponded to conserved sequences flanking the repetitive regions while secondary PCR primers were used to amplify IC3D7 and FC27 allelic variants. Positive DNA controls used (3D7, 7G8 and W2 strains) were obtained from the Malaria Research Reference Reagent Resource Centre. Two negative controls, one generated during DNA extraction and the other at the PCR stage, which consisted of only working reagents were included in all PCRs. Following amplification, secondary PCR products were resolved by electrophoresis on 2% ethidium-bromide stained agarose gels, visualized by ultraviolet and photographed using the AlphaDigidoc 1201 system (AlphaDigidoc, Alpha Innotech, San Leandro, CA). If there was no amplification for any allelic family, the electrophoresis was rerun using twice the quantity of the secondary PCR product. When the rerun did not show amplification, the primary and secondary PCRs were repeated using twice the quantity of DNA. When non-amplifications persisted after the repeat PCR, the amplification was classified as unsuccessful.

Methods for obtaining the entomological inoculation rates (EIR) for the study area during the period of this study have been previously described [7].

### Data interpretation and analysis

To determine the number of clones of malaria parasites (MOI) co-infecting an individual host, the number of distinct bands for all allelic variants was counted, and the total number represented an estimate of the minimum number of *P. falciparum* genotypes (parasites) carried by the host. For the MOI analysis, study subjects were divided into four age categories (0–4 years, 5–9 years, 10–17 years and 18–79 years) based on the presumed immunity of subject hosts and potential for use of preventive methods. Results were analysed using STATA version 10. One-way analysis of variance (ANOVA) was used to compare geometric mean MOIs between groups and trend analyses was carried out.

## Results

Four hundred and thirty-three (72.2%) of the 600 PCR amplifications were successful. The successful PCR amplifications obtained from the six bimonthly surveys (100 samples per survey round) were 71, 86, 92, 64, 82 and 38 in the respective months of Nov/Dec, Jan/Feb, Mar/Apr, May/Jun, Jul/Aug, Sep/Oct (Table 1). The only obvious reason which may have led to the lower PCR success rate in the sixth round can be attributed to the quality of samples received for analyses.

**Table 1:** MOI in the study cohort by month of survey

**Months**	**Number of samples tested**	**Successful amplifications**	**Total alleles**	Geometric mean MOI* for cohort	**95% confidence interval**	Geometric mean MOI* 0 – 4 years	**95% conf. interval**
Nov/Dec	100	71	131	2.03	1.83 – 2.25	2.13	1.89 – 2.39
Jan/Feb	100	86	230	2.83	2.53 – 3.12	3.08	2.72 – 3.48
Mar/Apr	100	92	279	3.39	3.06 – 3.75	3.37	2.85 – 3.98
May/Jun	100	64	131	1.96	1.75 – 2.20	2.18	1.83 – 2.60
Jul/Aug	100	82	193	2.56	2.3 – 2.85	2.40	2.04 – 2.83
Sep/Oct	100	38	51	1.36	1.25 – 1.48	1.67	1.44 – 1.94

* Overall P value for ANOVA test < 0.001.

### Distribution of MSP2 MOI and parasite density by month of year

The geometric mean MOI by month of year obtained for the study cohort as a whole and for the under five-year-old participants are shown in Table 1. The geometric mean parasite densities (GMPD) by month of year obtained for the cohort as a whole and for the under five–year-old participants are shown in Table 2. Children less than five years of age had a significantly (P < 0.001) high geometric mean MOI of 3.36 (95% CI: 2.85 – 3.97) in the March/April period compared to the September/October period when the geometric mean MOI was 1.67 (95% CI: 1.44 – 1.94) (Table 1). A similar trend in the monthly distribution of MOI was observed for the entire cohort with a significantly (P < 0.001) higher geometric mean MOI of 3.39 (95% CI: 3.06 – 3.75) in the March/April period, compared to the September/October period when the geometric mean MOI was 1.36 (95% CI: 1.25 – 1.48) (Table 1). In contrast, the highest and lowest GMPDs were not observed during these months. Instead, the highest and lowest GMPDs were observed in the May/June period and in the January/February period respectively (Table 2).

**Table 2:** Geometric mean parasite densities (GMPD) in the whole study cohort and in children 0 – 4 years of age by month of survey

**Months**	GMPD for cohort*	**95% Confidence interval**	**GMPD for** 0 – 4 years**	**95% Confidence interval**
Nov/Dec	4291	3421 – 5382	5217	3981 – 6837
Jan/Feb	2508	2054 – 3062	3148	2292 – 4324
Mar/Apr	3579	2893 – 4427	5193	3719 – 7252
May/Jun	4601	3730 – 5676	6978	5109 – 9530
Jul/Aug	3617	3038 – 4307	4572	3500 – 5971
Sep/Oct	3500	2880 – 4253	4756	3332 – 6790

* P values for ANOVA test = 0.001; ** P values for ANOVA test = 0.021.

### MSP2 MOI distribution by age

**Figure 1:**
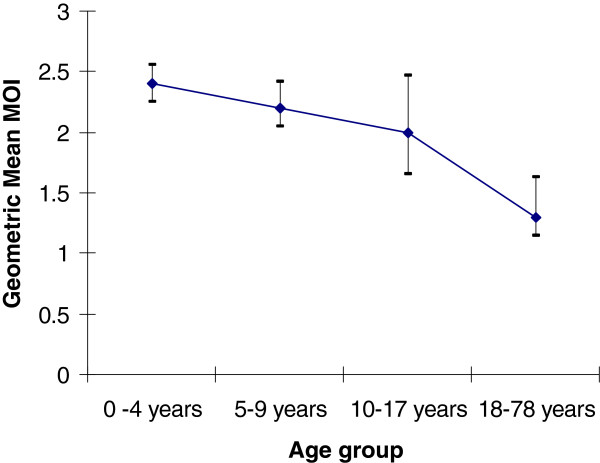
Trend of geometric mean MOI with age.

The youngest study participants were two babies both aged three weeks at enrolment. Their MOIs were two and five. The oldest study participant was a seventy-eight year old whose MOI was two. A summary of the geometric mean MOIs of age categories 0–4, 5–9, 10–17 and 18 years and above are shown in Table 3. Samples from children aged 0 to 4 years, 5 to 9 years and 10 to 17 years had geometric mean MOIs of 2.40 (95% CI: 2.25 – 2.55), 2.23 (95% CI: 2.05 – 2.42) and 2.03 (95% CI: 1.66 – 2.47) respectively during the year of observation (Table 3). No significant difference in MOI was observed between the 0 to 4 years age group and the 5 to 9 years age group (P = 0.972). Neither was the geometric mean MOI of those aged 0 to 4 years or of those aged 5 to 9 years significantly different compared to the 10 to 17 year old age group (P = 0.520 and P = 1.000 respectively). Adults, 18 years and above had a significantly lower (P < 0.001) geometric mean MOI of 1.37 (95% CI: 1.15 – 1.63), in comparison with the 0 to 9 years age group (Table 3). The overall effect of age on MOI was significant (p<0.001). The trend analysis suggested that geometric mean MOI decreased with increasing age. While the linear trend was significant (F-ratio=15.35, p<0.001), the curvilinear trend was not significant (F-ratio=3.21, p=0.07). The test also showed that geometric mean MOIs decreased across the age groups in a linear way and the error bar graph in Figure 1 strongly suggests this trend.

**Table 3:** Impact of age on MOI

**Age (years)**	**Number of samples tested**	**Successful amplifications**	**Total alleles**	Geometric mean MOI*	**95% confidence interval**
0 – 4	308	243	550	2.40	2.25 – 2.55
5 – 9	229	155	393	2.23	2.05 – 2.43
10 – 17	42	26	62	2.03	1.66 – 2.47
18 – 78	21	9	10	1.37	1.15 – 1.63

* P value for ANOVA test < 0.001.

### Monthly prevalence of MSP2 alleles and intensity of malaria transmission

**Figure 2:**
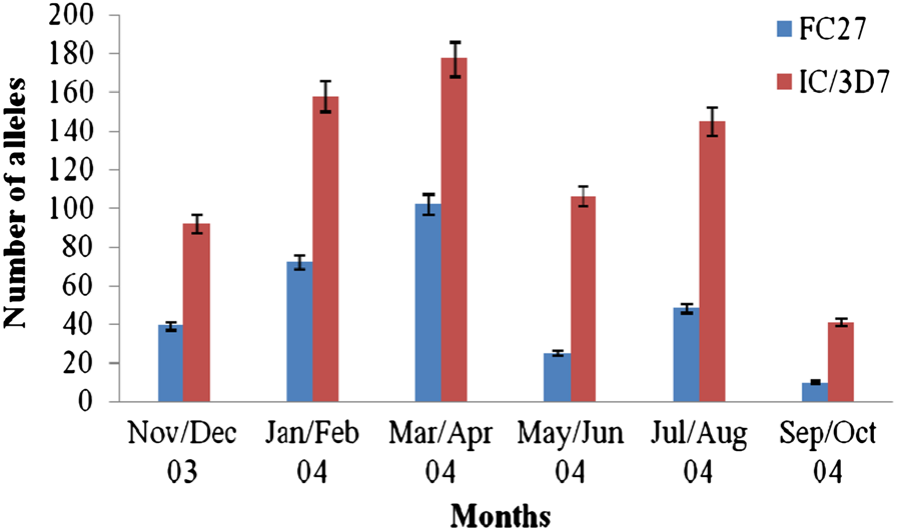
Bimonthly distribution of MSP2 alleles in study cohort.

**Figure 3:**
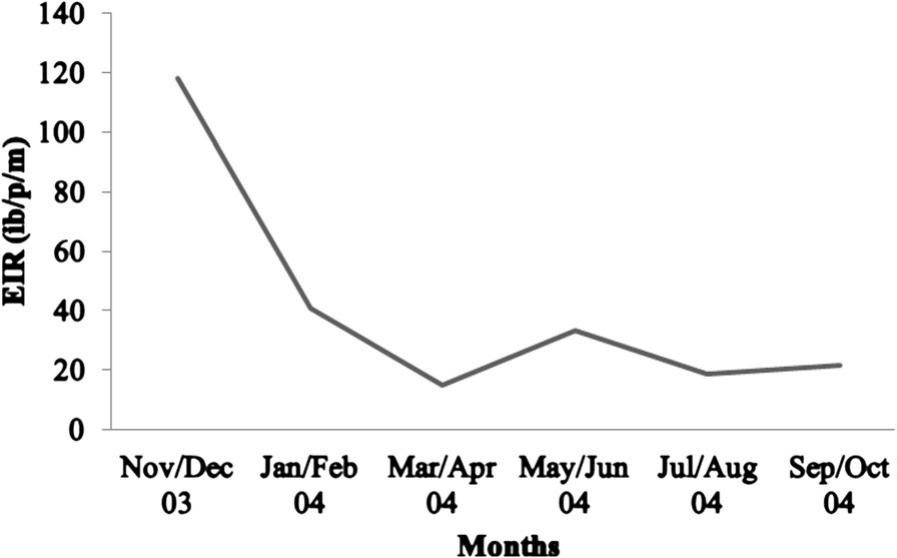
Bimonthly prevalence of EIR within study area.

IC/3D7 strains were detected more frequently than FC27 strains throughout the year. In the March/April period when parasites carrying either allele were detected most frequently, the ratio of the prevalence of IC/3D7 strains compared to the FC27 was about 2:1 (Figure 2). The EIR was lowest in the March/April period (when both MSP2 alleles were detected most frequently) and low again in the September/October period when both MSP2 alleles were detected the least frequently (Figures 2 and 3). The EIR was highest between November/December.

## Discussion

In the Kintampo area, the rainy season spans from March to November with two peaks. The major peak season between March and July and the minor peak season September and November. This study showed that MOI was not influenced by the GMPD and vice versa. While MOI was highest in March/April GMPD was highest in May/June. Significant correlation between parasite density and MOI have been demonstrated in infants and children (1–2 years of age) but not in older individuals [20,21] and high MOIs have been shown to be characteristic of low-level chronic parasitaemia in individuals older than two years [22]. Therefore, the lack of an association between the parasite densities and MOI among the whole cohort is to be expected. Results of this study however suggest that in this study area, the potential of the asymptomatic population to harbour diverse *P*. *falciparum* strains was highest between March and June (major wet season). Malaria control needs to be stepped-up and strictly adhered to during these months. Visitors need to make prophylaxis a priority.

The MOI decreased significantly with increasing age. This age-effect was however most marked in subjects older than 17 years. This finding agrees with a study in Senegal where the number of genotypes decreased with age in those aged 15 and above [10]. That children aged between 0 and 9 years seemed to be the drivers of parasite diversity in this study involving an asymptomatic population is to be expected as noted in Tanzanian children in whom MOIs peaked in 3–7 year olds [23]. These findings are in agreement with other studies, which have shown correlations between parasite density and MOI in infants and young children but not in older individuals [10,24]. The existence of a positive association between high childhood MOI and low adulthood MOI in asymptomatic subjects is in keeping with the concept that multiple strains of parasite in early life is necessary to produce immunity in adults.

It is reported that an increase in transmission intensity generally correlates with a progressive increase in the average number of malaria parasite clones [8,9]. In this study, a clear relationship between asymptomatic MOI and transmission (estimated using the EIR) was not observed. Whereas the EIR was lowest during the period of highest MOI (March/April), relatively low EIRs were still recorded during the period of lowest MOI (September/October). The observation of low transmission during the period of highest MOI could be due to the simultaneous occurrence of low parasite densities. Nevertheless, these findings suggest that the transmission of parasites was independent of the number of malaria parasite clones therefore interventions to reduce transmission may have little effect on the parasite diversity among the asymptomatic cohort.

## Conclusion

This study has defined the influence of age and season on MOI in a central area of Ghana. These findings provide knowledge upon which the impacts of interventions (including the RTS, S/AS01 candidate malaria vaccine) introduced since this baseline study was carried out will be analysed in future investigations involving asymptomatic individuals.

## Abbreviations

ANOVA: Analyses of variance; DNA: De-oxy-ribonucleic acid; EIR: Entomological inoculation rate; GMPD: Geometric mean parasite density; MOI: Multiplicity of infection; MSP2: Merozoite Surface Protein 2; PCR: Polymerase chain reaction.

## Competing interests

The authors declare that they have no competing interest.

## Authors’ contributions

SOA conceptualised the idea, wrote the proposal, secured funding and was the principal investigator. MA, DO and DD carried out sample collections; GD performed data management; AAB, CB and DD carried out laboratory analyses; AAB and CB drafted the initial paper; KPA and SOA supervised data collection, analyses interpretation and write up and MW, BG advised on the design and implementation of the study and also reviewed and finalised this paper for publication. All authors reviewed, read and approved the final manuscript.
